# Wettability modified nanoporous ceramic membrane for simultaneous residual heat and condensate recovery

**DOI:** 10.1038/srep27274

**Published:** 2016-06-07

**Authors:** H. W. Hu, G. H. Tang, D. Niu

**Affiliations:** 1 MOE Key Laboratory of Thermo-Fluid Science and Engineering, School of Energy and Power Engineering, Xi’an Jiaotong University, Xi’an 710049, P.R. China

## Abstract

Recovery of both latent heat and condensate from boiler flue gas is significant for improving boiler efficiency and water conservation. The condensation experiments are carried out to investigate the simultaneous heat and mass transfer across the nanoporous ceramic membranes (NPCMs) which are treated to be hydrophilic and hydrophobic surfaces using the semicontinuous supercritical reactions. The effects of typical parameters including coolant flow rate, vapor/nitrogen gas mixture temperature, water vapor volume fraction and transmembrane pressure on heat and mass transfer performance are studied. The experimental results show that the hydrophilic NPCM exhibits higher performances of condensation heat transfer and condensate recovery. However, the hydrophobic modification results in remarkable degradation of heat and condensate recovery from the mixture. Molecular dynamics simulations are conducted to establish a hydrophilic/hydrophobic nanopore/water liquid system, and the infiltration characteristics of the single hydrophilic/hydrophobic nanopore is revealed.

A large amount of energy consumed comes from hydrocarbon fuel combustion, and water vapor is one of the main combustion products. Especially in gas fired boiler, up to 20% volume fraction of water vapor in combustion products can be generated. The residual heat and the water recovery from the flue gas generated in hydrocarbon fuel combustion can improve the thermal efficiency and represent a real new source of potable water. The condensation heat transfer in various heat exchangers is one of the traditional technologies for heat and water recovery from the flue gas^1^,^2^,^3^,^4^,^5^,^6^,^7^. However, the corrosion during the recovery limits its use due to the presence of acid pollutant, such as SO_x_ and NO_x_. Another alternative is the chemical or physical adsorption of water by a liquid or solid desiccant^8^. In this case, the cost, the regeneration of adsorbent and the quality of water are the main disadvantages.

The membrane condenser as a novel heat exchanger can overcome the weaknesses of conventional technologies in residual heat and water recovery. Actually, water vapor transport across membranes has a wide and important utilization in many industrial processes, such as desalinization of sea water, drying of natural gas, and flue gas dehydration^9^,^10^,^11^,^12^. Membrane technology is an attractive, energy efficient alternative for molecular separations because of its high efficiency, reliability, and compact volume. It allows the selective removal of water vapor from mixture and can produce water with high purities. In addition, the membrane heat exchanger may have higher heat recovery efficiency than the conventional one because simultaneous mass and heat transfer occurs in the membrane heat exchanger.

There has been a growing number of studies on the new condensation technology of membrane. Sijbesma *et al*.^13^,^14^ presented a composite hollow fiber membrane with a top layer of SPEEK, for the removal of water vapor from flue gas, and it had extremely high separation factors and fluxes. In their field test with artificial exhaust gas, the developed membranes continuously removed water vapor of 0.6–1 kg/m^2^h. And the average water vapor removal rate of 0.2–0.46 L/m^2^h was obtained during a continuous long-term field test of 5300 h under real exhaust gas conditions. A membrane condenser for the selective recovery of water vapor from industrial gases was developed^15^,^16^,^17^. It utilized the hydrophobic property of porous membranes with microscale pore size to hold the water droplets present in the mixture. Macedonio *et al*.^15^ proposed membrane technology using hydrophobic membranes for the separation and recovery of water vapor from industrial processes. Their experimental results indicated that 20% water recovery was achieved with temperature reduction below 5 °C. Both asymmetric microporous spongelike ethylene-chlorotrifluoroethylene copolymer (ECTFE) flat sheet membranes and commercial polyvinylidene fluoride (PVDF) hollow fiber membranes were tested by varying the feed temperature and feed flow rate^16^. PVDF microporous hydrophobic fibers assembled in a module were tested by Brunetti *et al*.^17^ It was found that 25% water vapor contained in the feed was recovered at the temperature difference of 8 °C and the ratio of the feed flow rate with the membrane area was a fundamental parameter to be taken into account in the design of the membrane unit. Zhang *et al*.^18^ affirmed that the permeability ratio of water to air ranged from 460 to 30000 for such hydrophilic membranes. In other words, gases other than water vapor could hardly permeate through these membranes.

Gas Technology Institute (GTI) in the United States developed a new membrane technology of transport membrane condenser (TMC) based on a nanoporous ceramic separation membrane^19^,^20^, which was employed to extract a portion of water vapor and its latent heat from the flue gas and return the recovered water and heat to the steam cycle. Then, Bao *et al*.^21^ experimentally studied this phenomena for both nanoporous membrane tube bundle and impermeable stainless steel tube bundle with the same characteristic dimensions, and they found that the convective condensaiton performance of the membrane tube bundle was 50–80% higher than that of the impermeable stainless steel tube bundle. Through the theoretical thermodynamic analysis of mass and heat transfer in the membrane condensation system, Yan *et al*.^22^ suggested that the heat recovery of membrane condensation improved dramatically with the increase of the inlet gas temperature. Wang *et al*.^23^ employed a tubular ceramic membrane as the condenser for simultaneous water and heat recovery from the air/vapor mixure, and up to 60% water recovery and up to 85% heat recovery were both achieved.

Although reserches have been reported on the condensation technology of membrane recently, there was no investigation of the wettability modificaiton on the NPCM. In this work, the potential of the wettabilty modified NPCM was explored for simultaneous residual heat and condensate recovery from vapor/nitrogen mixture. The NPCMs were modified to be hydrophilic and hydrophobic surfaces using the semicontinuous supercritical reactions. We experimentally investigated and compared the performance of heat and mass transfer on original, modified hydrophilic and hydrophobic NPCM tubes. Condensate bebaviors on the surfaces of original and modified hydrophilic and hydrophobic NPCMs were observed. Parametric studies were also carried out by varying cooling water flow rate, vapor/nitrogen mixture temperature, water vapor volume fraction and transmembrane pressure. In addition, a computational model of a single nanopore with different wettability was established using the molecular dynamics simulations to reveal the infiltration characteristics of liquid molecules into hydrophilic/hydrophobic nanopores.

## Results

### Wettability and morphology of fabricated NPCMs

**Figure 1:**
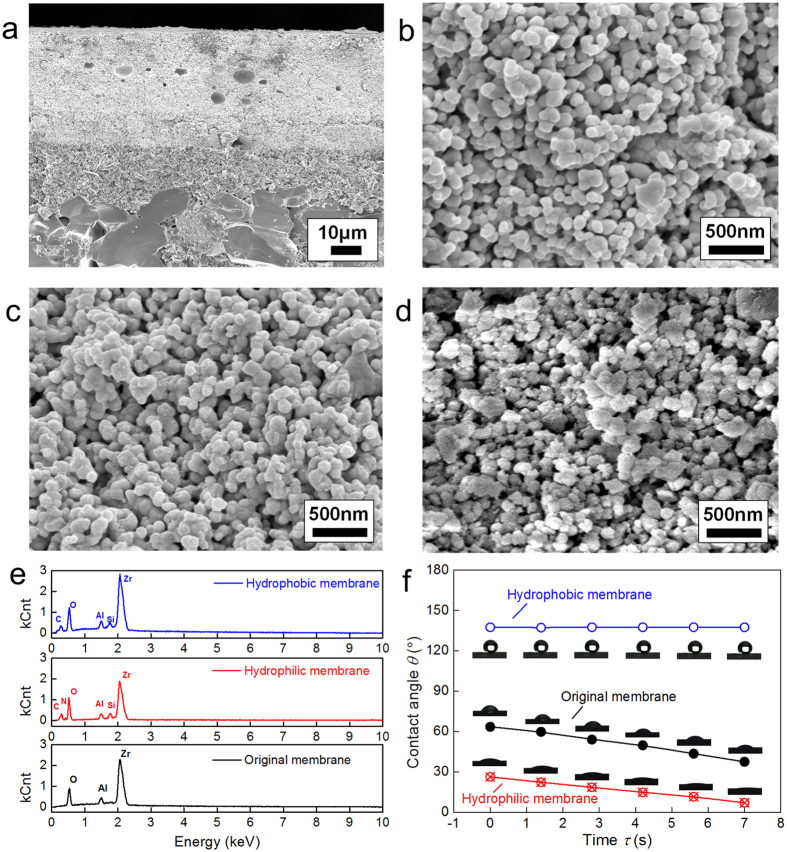
Microtopography characterization of NPCM. (**a**) FESEM image of porous membrane tube cross-section coated by the nanoporous layer (1000 times magnification). (**b**) FESEM image of the original NPCM layer (35000 times magnification). (**c**) FESEM image of the NPCM layer with hydrophilic modification (35000 times magnification). (**d**) FESEM image of the NPCM layer with hydrophobic modification (35000 times magnification). (**e**) Electron dispersive X-ray spectroscopy (EDS) of original, modified hydrophilic and hydrophobic NPCM layers. (**f**) Measured contact angles on original and modified hydrophilic and hydrophobic NPCM surfaces over time.

The NPCMs were modified to be hydrophilic and hydrophobic using the semicontinuous supercritical reactions in the custom built apparatus (The details can be found in the Methods Section). Figure 1a shows the microstructure image of porous membrane tube cross-section coated by the nanoporous layer obtained by a field emission scanning electron microscope (FESEM) (Hitachi-SU8010, Japan). From Fig. 1a it is found that the membrane tube wall consists of three layers: a separation layer, an intermediate layer and a substrate. The separation layer, i.e., nanoporous membrane layer is on the outer tube side of the tubular membrane. The tube samples (outer diameter of 10 mm, inner diameter of 6 mm, length of 250 mm, and average pore size of seperation layer of 4 nm and 10 nm) are provided by Jiangsu Nanjing Ayuqi Sci-tech Co. Ltd. This graded structure was used for both polymeric and ceramic nanoporous separation membranes to achieve high separation ratio with minimal resistance to flux of the permeating species^21^.

Figure 1b–d show the FESEM images of the NPCM layers with and without wettability modification. It is seen that the original and hydrophilic modified nanoporous membranes are accumulated by nanoscale spherical particles and the hydrophilic modification does not affect the morphology of the nanoporous membrane significantly. And tiny burrs appears on nanoscale spherical particles through the hydrophobic modificaiton. To provide appropriate spectroscopic characterization data, in addition, the original and modified membranes have been analyzed and confirmed using the electron dispersive X-ray spectroscopy (EDS). As shown in Fig. 1e, the characteristic peaks of C and Si can be observed on the hydrophobic membrane surface, and the characteristic peaks of C, N and Si can be observed on the hydrophilic membrane surface, comprared to the original membrane surface. The contact angles of deionized water droplets on the original, modified hydrophilic and hydrophobic membrane surfaces are measured using a contact angle measurement apparatus (Powereach JC2000D5, China) with uncertainty of ±0.25°. The measurements are shown in Fig. 1f. We can see that the original membrane also behaves hydrophilic. Once the droplets contact the original and modified hydrophilic surfaces, the measured contact angles are 63.25° and 26.25°, respectively. Due to both the surface tension and capillarity, droplets on the original and modified hydrophilic surfaces can infiltrate into the pores, and the contact angles gradually decrease over time. The static contact angle of the hydrophobic nanoporous membrane surface is 137.5°, and hardly changes over time.

### Condensate recovery performance

**Figure 2:**
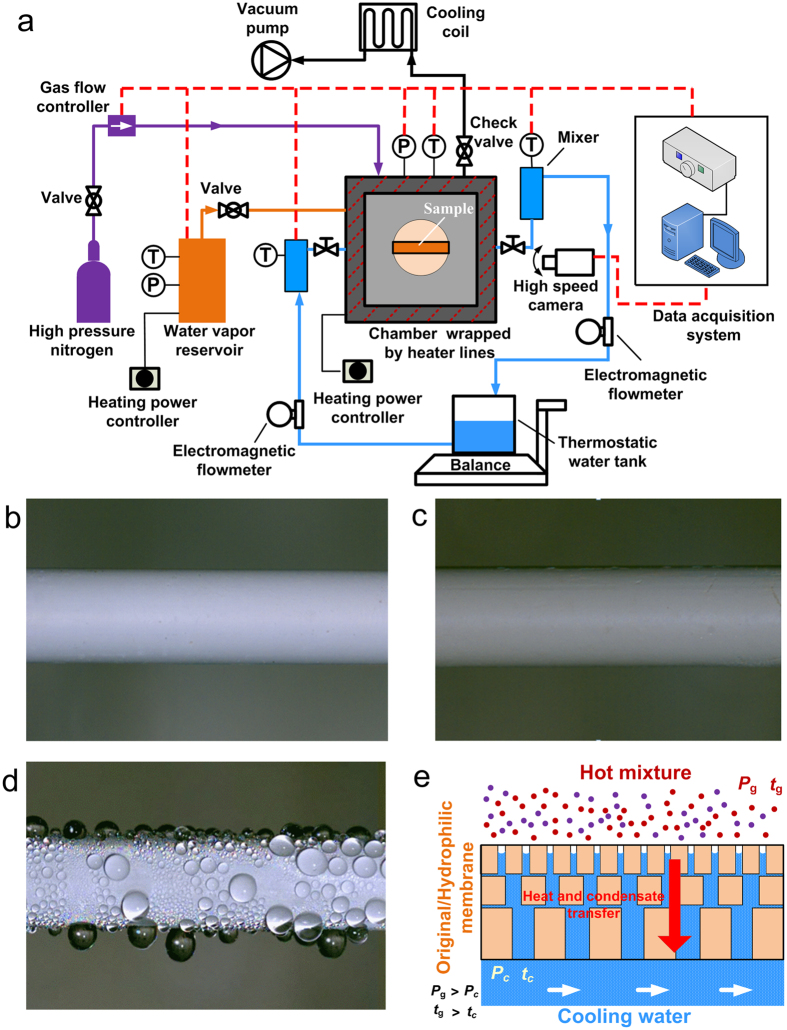
Test setup and experimental images of condensation. (**a**) Schematic diagram of the condensation heat transfer experimental apparatus. (**b**,**c**) No condensate on the original and modified hydrophilic NPCM tubes during condensation. (**d**) Dropwise condensation on the hydrophobic NPCM tube. (**e**) Schematic of heat and mass transfer across hydrophilic NPCMs.

The NPCM tubes with and without wettability modification were characterized during the condensation heat transfer using a custom built apparatus as schematic in Fig. 2a. The details of the apparatus and testing procedures can be found in the Methods Section. The condensation experimental system was made up of the following parts: condensing chamber, coolant circulating loop, water vapor generator, high pressure nitrogen supply and data acquisition and control system.

Figure 2b–d show typical images of condensation behaviors extracted from high-speed imaging duing the condensation heat transfer on the surfaces of original, modified hydrophilic and modified hydrophobic NPCM tubes. It is seen that there is no condensate on the surfaces of both original and modified hydrophilic NPCMs, nevertheless the dropwise condensaiton forms on the surface of the modified hydrophobic NPCM. Figure 2e illustrates the heat and mass transfer mechanisms across the original and modified hydrophilic nanoporous membranes. When the temperature of the ceramic membrane tube surface is lower than the dew point temperature, which depends on the partial pressure of water vapor, the vapor/nitrogen mixture passes across the tube wall and the condensation of water vapor takes place in the pores of the membrane layer or on the surface. The condensate is then convected away from the surface under a certain transmembrane pressure difference. A curved meniscus forms in the hydrophilic membrane pores when the capillary condensation occurs in the membrane condensation. For the hydrophobic NPCM, the condensate cannot overcome the infiltration pressure into the hydrophobic nanopores, so it is difficult for the condensate to be carried away by the cooling water flowing inside the tube and therefore the droplets form on the hydrophobic membrane surface.

**Figure 3:**
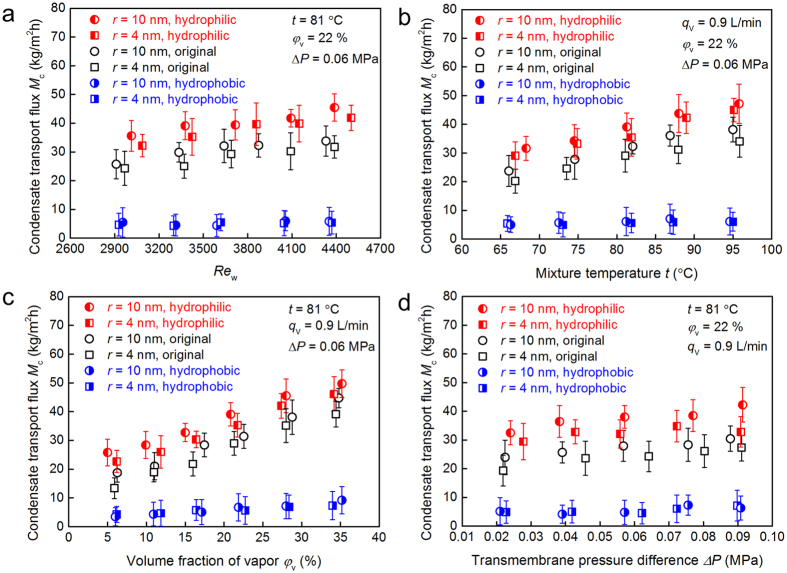
Condensation experimental measurement of condensate transport flux. (**a**) Experimental condensate transport flux across the membrane against Reynolds number of the cooling water. (**b**) Experimental condensate transport flux across the membrane against the vapor/nitrogen mixture temperature. (**c**) Experimental condensate transport flux across the membrane against the water vapor volume fraction in the mixture. (**d**) Experimental condensate transport flux across the membrane against the transmembrane pressure difference.

Heat and mass transfer occurs simultaneously across the NPCM. In this study, we define the condensate transport flux to evaluate the condensate recovery performance, *M*_c_ = Δ*m*_c_ / (Δ*τ A*_o_), where Δ*m*_c_ is the measured mass change of the liquid water during a period of time Δ*τ*, and *A*_o_ is the outside area of tested NPCM tube without considering the effect of porous surface. Figures 3a–d show the effects of typical parameters, i.e., the cooling water flow rate, vapor/nitrogen mixture temperature, water vapor volume fraction and transmembrane pressure difference on the condensate transport flux of NPCMs with and without wettability modification.

The Reynolds number of the cooling water inside the tube is defined as *Re*_w_ = 4 *q*_V_ / (π *d*_i_ *ν*), where *d*_i_ is the internal diameter of the tested tube, *q*_V_ and *ν* are the volume flow rate and kinematic viscosity of the cooling water. Both Reynolds number and mixture temperature have important effect on the condensate transport flux as presented in Fig. 3a,b, respectively. It is seen that the condensate transport flux increases with the increase of the coolant flow rate and the mixture temperature. As the mixture temperature increases from 65.8 °C to 95.9 °C, the condensate transport fluxes of the original and modified hydrophilic NPCMs grow almost linearly. The main reason is that the high driving force resulted from the higher water vapor partial pressure on the gas side at a higher temperature can lead to higher mass flux^23^,^24^. Compared with the original membrane, the condensate transport flux of modified hydrophilic membrane increases by 32%. The volume fraction of water vapor has a significant effect on the condensate transport flux across the membrane condenser, as shown in Fig. 3c. As the volume fraction of water vapor increases, the condensate transport flux improves dramatically due to the increase of driving force of mass transfer. Under the condition of the vapor volume fraction of 4.9–35.2%, the condensate transport flux of the modified hydrophilic NPCM increases by 17–69%, compared with the original membrane with average pore size of 10 nm. The relationship between the condensate transport flux and the transmembrane pressure difference is shown in Fig. 3d. It seems that the condensate transport flux does not change much as the transmembrane pressure difference increases from 0.021 MPa to 0.092 MPa. In addition, we can see that the large membrane pore size can produce higher permeability with respect to the condensate transport flux. From Fig. 3 it is also found that the parameters of cooling water flow rate, vapor/nitrogen mixture temperature, water vapor volume fraction and transmembrane pressure difference have negligible effect on the condensate transport flux of the modified hydrophobic NPCM. The modified hydrophobic NPCM demonstrates approximately 70% degradation of the condensate transport flux compared with the original membrane. For modified hydrophilic NPCMs, the condensation origins from two sources: one is the cooling effect of the cold surface, and the other is capillary condensation in the hydrophilic pores of the membrane. Note that the capillary condensation is important here because the water vapor can condense inside the pores of the membrane. Furthermore, the capillary condensation dominates the mass transfer mechanism when a hydrophilic membrane pore size is in the range of 2–50 nm^25^,^26^. During initial adsorption stage, water vapor molecules are more easily adsorbed on a high free energy hydrophilic surface, in comparison to the hydrophobic surface. In addition, the degrading of condensate transport flux of modified hydrophobic NPCMs is attributed to the block of condenste transport in the membrane due to the gas-liquid interface caused by the hydrophobicity.

### Condensation heat transfer performance

The condensation heat fluxes are measured to evaluate the heat recovery performance on the three types of tubes with different wettability: the original, the modified hydrophilic and the modified hydrophobic NPCM tubes. Condensation heat flux *q*″ is determined by the expression of *q*″ = (*ρ*(*t*_out_) *q*_V,out_ *h*_out_−*ρ*(*t*_in_) *q*_V,in_ *h*_in_)/*A*_o_, where *h*_in_, *h*_out_, *ρ*(*t*_in_) and *ρ*(*t*_out_) are the specific enthalpies and densities at inlet and outlet cooling water temperatures, respectively, and *q*_V_ is the measured volume flow rate of cooling water. The outlet flow rate of cooling water is increased due to the extra mass transfer across membrane compared with the inlet flow rate.

**Figure 4:**
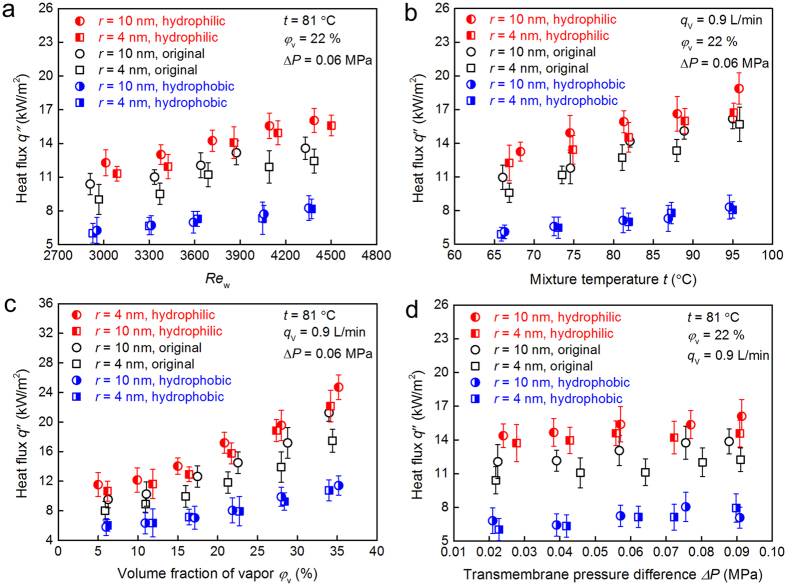
Condensation heat transfer performance as function of typical parameters. (**a**) Heat flux across the membrane against Reynolds number of the cooling water. (**b**) Heat flux across the membrane against the vapor/nitrogen gas mixture temperature. (**c**) Heat flux across the membrane against the water vapor volume fraction in the mixture. (**d**) Heat flux across the membrane against the transmembrane pressure difference.

The measured condensation heat fluxes across the original, modified hydrophilic and hydrophobic NPCMs as a function of typical parameters of the cooling water flow rate, vapor/nitrogen mixture temperature, water vapor volume fraction and transmembrane pressure difference are presented in Fig. 4a–d, respectively. In Fig. 4a,b, the heat flux has similar growth trends of the condensate transport flux, since the condenation heat transfer across the membranes is closely associated with the mass transfer. A high flow rate of cooling water or high vapor/nitrogen mixture temperature produces a large temperature difference between the hot mixture and the membrane tube surface. Thus, the condensation heat transfer performance is enhanced due to the increase of driving force of heat transfer. The heat flux of the modified hydrophilic NPCM increases approximately by 26% compared with the original NPCM. Figure 4c shows that the measured heat flux improves significantly, as the volume fraction of water vapor becomes larger. When the water vapor concentration difference between the mainstream and the region near the surface becomes large, more water vapor in the mixture condenses. As the water vapor concentration increases, large volume fraction of water vapor results in high vapor partial pressure, and then results in large driving force of mass transfer. In addtion, the capillary condensation on the original and modified hydrophilic nanoporous membranes cannot add extra thermal resistance of liquid phase, and large amount of latent heat is released by the water vapor. In comparison to the original NPCM, the heat flux of modified hydrophilic NPCM increases by 26–34% as the vapor volume fraction increases from 4.9% to 35.2%. As shown in Fig. 4d, the heat fluxes of original, modified hydrophilic and modified hydrophobic NPCMs have little improvement in the range of the transmembrane pressure difference of 0.021–0.092 MPa. In comparison to the original NPCM as shown in Fig. 4a–d, the condensation heat transfer performance of modified hydrophobic membrane exhibits 24–40% degradation. This is mainly because the hydrophilic NPCMs can adsorb the water vapor more easily and the capillary condensation phenomenon appears, as mentioned above. Based on the pore capillary condensation mechanism, Kelvin equation^27^ (ln (*P* / *P*_0_) = 2 *γ V*_m_ / (*r R T*), where *P* is the actual vapor pressure, *P*_0_ is the saturated vapor pressure, *γ* is the surface tension, *V*_m_ is the molar volume of the liquid, *R* is the universal gas constant, and *T* is temperature) indicates that the vapor pressure of concave surface is lower than that of a flat or non-curved surface. So the pore capillary condensation can occur even if the partial pressure of vapor is below the saturation pressure at the same temperature, especially for the membrane pore in nanoscale. The mass transfer across the membrane has a significant effect on condensation heat transfer performance. That is to say, the presence of the adsorption property and capillary condensation is attributed to increasing the condensation rate, therefore, the condensation heat transfer performance is enhanced.

## Discussion

**Figure 5:**
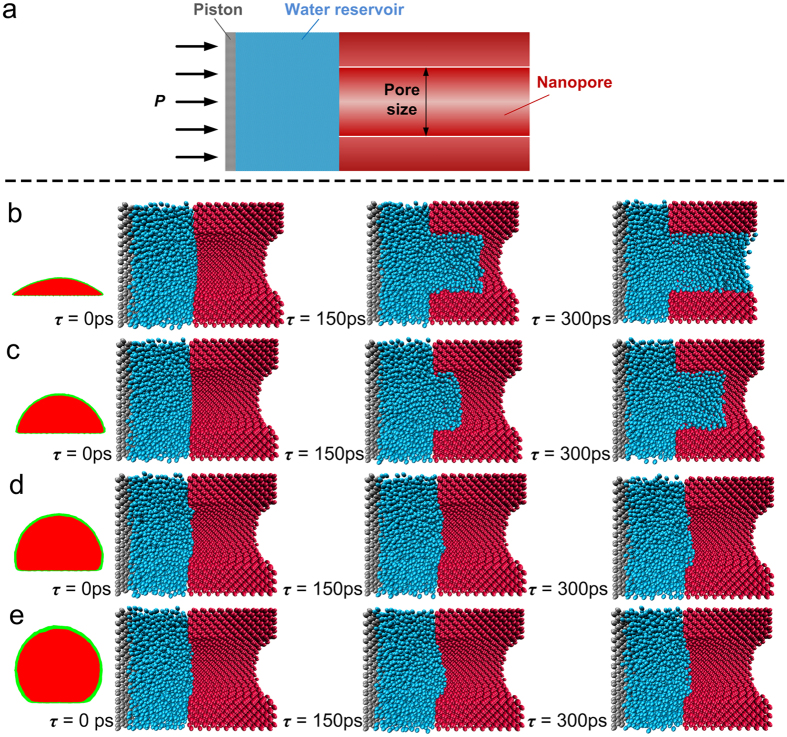
The geometric model of computational nanopore/water liquid system and infiltration performance of nanopores with different wettability. (**a**) The MD computational model of nanopore/water liquid system. One end of the nanopore is inserted into a water reservoir, the other end is open, and an external pressure is applied on the mobile piston. (**b**–**e**) Infiltration characteristics of water molecules into hydrophilic and hydrophobic nanopores under an external pressure of 0.1 MPa.

To better understand and interpret the experimental results, we employed the molecular dynamics (MD) simulations to investigate the water infiltration behavior in a nanopore of 4 nm pore size for different surface wettability. All the present simulations are based on the LAMMPS package^28^. The monatomic water model^29^,^30^ described by a Stillinger-Weber potential^31^ is employed. We adopted a diamond crystal structure of silicon with a lattice constant 5.4 Å to construct the nanopore by deleting the atoms located at the center of the bulk with a diameter of 4 nm. The solid wall is treated as a rigid body and the wall atoms are just fixed in the initial position during the simulation. The 12-6 LJ potential, 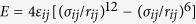, is adopted to describe the interaction between the solid wall and the water molecules using different energy parameters to cover the surface property from the hydrophobic to the hydrophilic as shown in Fig. 5. The corresponding *ε*_ij_ in Fig. 5b–e is 0.4 kcal/mol, 0.3 kcal/mol, 0.2 kcal/mol, 0.1 kcal/mol, respectively, and *σ*_ij_ is 3.4 Å in all cases. Considering the experimental operating conditions, the pressure difference between the internal cooling water and external vapor/nitrogen mixture is a maximum of 0.1 MPa, so we control the same pressure difference by applying a uniform force on the left piston as shown in Fig. 5a. Note that the water molecules are all outside of the nanopore initially. However, for the same loading, the nanopore shows different infiltration process depending on the surface wettability. For the hydrophilic nanopore, we can see that the water molecules enter the nanopore smoothly. Furthermore, comparing Fig. 5b with 5c, we can see that the higher hydrophilicity will lead the water molecules to move into the nanopore faster. On the contrary, for the two hydrophobic nanopores shown in Fig. 5d,e, the pressure difference of 0.1 MPa is still too small to pressurize the water into the nanopore. The simulation results are consistent with the present observation that the droplets occur on the modified hydrophobic surface rather than into the ceramic membrane tube. Conversely, no condensate is observed outside the original and modified hydrophilic NPCM tubes during condensation, which indicates that the condensate passes through the membrane and is carried away by the cooling water.

**Figure 6:**
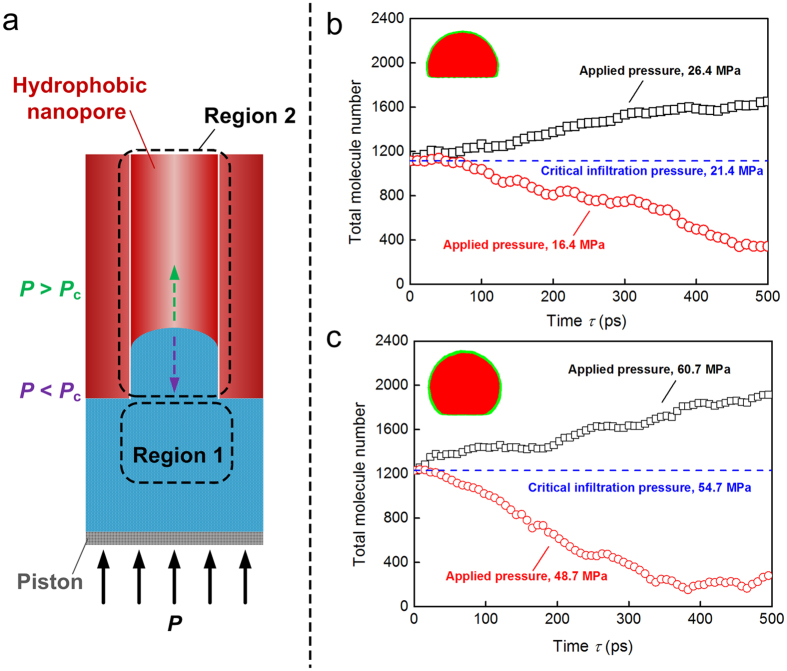
Critical infiltration pressure of hydrophobic nanopores. (**a**) Partially filled hydrophobic nanopore/water liquid system. (**b**,**c**) Effects of hydrophobicity on the critical infiltration pressure.

Based on the simulation results shown in Fig. 5, we have confirmed that the experimental operating pressure cannot pressurize the condensate into the hydrophobic nanopore. So another initial structure shown in Fig. 6a is built to accurately calculate the infiltration pressure *P*_c_, the critical pressure to achieve water entering the hydrophobic nanopore. All the simulations are performed in two stages. In the first stage, the piston is fixed during the simulation and the nanopore is partially filled until the water reaches equilibrium state. Then we calculate the critical pressure in Region 1 for two hydrophobic nanopores. In the second stage the piston is unfixed and the uniform force above or below the critical infiltration pressure is applied on the piston. The water molecule number in Region 2 is calculated for different loadings, as shown in Fig. 6b,c. From the simulation results obtained in the first stage, the critical infiltration pressures are 21.4 MPa and 54.7 MPa represented by the blue dotted line in Fig. 6b,c, respectively. This means that the nanopore surface with well hydrophobicity results in a large critical infiltration pressure which is qualitatively consistent with the prediction of Young-Laplace equation. From Fig. 6b,c, when the applied pressure is larger than the critical infiltration pressure, the water continues to enter the hydrophobic nanopore. On the contrary, the water will flow out of the hydrophobic nanopore if the applied pressure is less than the critical infiltration pressure.

## Methods

### Wettability modification for nanoporous membranes

**Figure 7:**
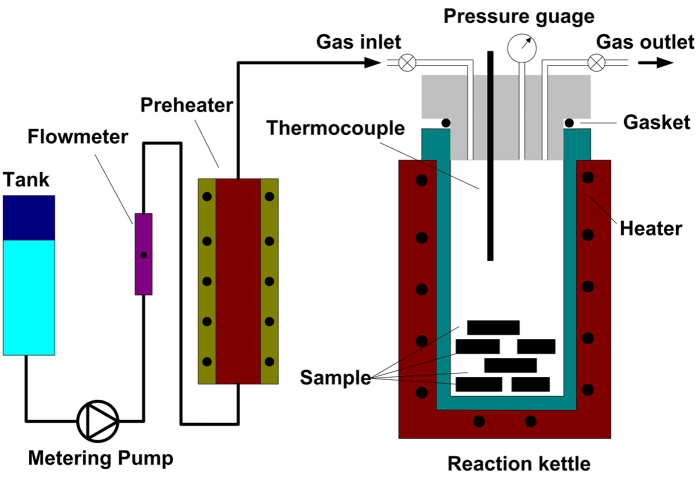
Schematic experiment apparatus of the semicontinuous supercritical reactions.

The modified hydrophilic and hydrophobic NPCMs were obtained from the semicontinuous supercritical reactions in the custom built apparatus, which was composed primarily of reaction kettle, electric preheater, high pressure metering pump and vacuum pump as schematic in Fig. 7. Prior to the wettability modification, the samples of original tubes were cleaned in an ultrasonic bath with deionized water for 30 min at room temperature. The cleaned samples were placed in the reaction kettle. Switch on the power, and the temperature inside the reaction kettle and the electric preheater was kept at 250 °C. Then, the vacuum pumping operation kept for 30 min using a rotary vane vacuum pump (First FX16, China). Hexane was continually pumped into the reaction kettle through the high pressure metering pump until the pressure reaches 3 MPa. For the hydrophobic modification, the hexane solution of 10 wt% dimethyl dimethoxy silicane ((CH_3_)_2_Si(CH_3_O)_2_, Aladdin) and 5 wt% trimethylmethoxysilane (C_4_H_12_OSi) was subsequently injected into the reaction kettle. The hexane solution of 10 wt% aminopropyltrimethoxysilane (C_9_H_23_NO_3_Si, Aladdin) was subsequently injected into the reaction kettle for hydrophilic modification. With the continuous injection of modified chemical reagent steam, the pressure in the reaction kettle was raised up to 5 MPa. The reaction between the samples and the modified chemical reagent steam kept for 2 hours at constant temperature of 250 °C and constant pressure of 5 MPa. The wettability modification progress was completed after the procedure of the isothermal pressure relief. To achieve higher hydrophilicity, besides, the samples after the hydrophilic modification were put in boiling water for 6 hours to make an oxygen radical hydrolysis.

### Condensation heat transfer performance test

To evaluate the condensation heat transfer, the sample of NPCM tube was plugged into a condensing chamber made of stainless steel. The resistive heater lines were wrapped around the exterior of the condensing chamber walls to prevent vapor condensation on the inner walls. The temperature in the chamber could maintain at the expected temperature of the vapor side using a voltage regulator, which was the output power controller of resistive heater lines. A water reservoir, which was connected to the condensing chamber via a vapor valve, was heated to saturated temperature to provide vapor. Prior to the condensation experiment, the air in the chamber was fully evacuated by a rotary-vane vacuum pump (First FX16, China). The vacuum condition in the condensing chamber was always monitored by the pressure transducer (Tecisis P3276, Germany) with the full scale of 0–0.25 MPa. A check valve was attached onto the chamber to prevent any backflow of air after turning off the vacuum pump. The nitrogen gas treated as noncondensable gas was supplied by high pressurized nitrogen gas cylinder with a pressure reducing valve. The nitrogen gas flowing into the condensing chamber was monitored and controlled by the gas mass flow controller (Sevenstar CS200A, China). During the condensation heat transfer test, the heat released from the vapor/nitrogen mixture was taken away by the forced flow of cooling water inside the tested tube. The inlet and outlet cooling water flow rates were measured by electromagnetic flowmeters (Rosemount 8732A, USA) with same measurement range and uncertainty. Especially, to obtain the bulk temperature of the cooling water accurately, the mixers assembled by baffles with holes alternately near the center and perimeter were installed at the inlet and outlet^32^. All the measured temperature data were collected by T-type thermocouples. Throughout the experiments, the condensing chamber pressure and temperature were continuously monitored. The bulk temperatures of the cooling water at the inlet and outlet were measured to determine the heat transfer rate. The cooling water temperature was maintained using a thermostatic water tank, which was placed on a mass balance (YP50K-1, China) to monitor the mass change Δ*m*_c_ of the water during a period of time Δ*τ*. Once the experimental condition reaches steady state, both the mass and temperature data of cooling water in thermostatic water tank were recorded repeatedly for 30 min at a time interval of 3 min. To visually record the condensation behavior, a high speed camera (Phantom Miro M110, USA) was placed in line with the viewing window on the condensing chamber. All the thermocouples were calibrated against a platinum resistance thermometer in a high precision constant temperature bath (Julabo F26, Germany). The measured experimental data including the pressure, thermocouple reading, noncondensable gas content and flow rate of the cooling water were collected by a data acquisition system (Keithley 3706A, USA). All the measurements were conducted under steady conditions. The maximum uncertainty of the cooling water Reynolds number is ±1.7%, the maximum uncertainty of vapor volume fraction in the mixture is ±1.8 %, the maximum uncertainty of the condensate transport flux is ±3.3%, and the maximum uncertainty of the condensation heat flux is ±7.0%.

## Additional Information

**How to cite this article**: Hu, H.W. *et al*. Wettability modified nanoporous ceramic membrane for simultaneous residual heat and condensate recovery. *Sci. Rep*. **6**, 27274; doi: 10.1038/srep27274 (2016).

## References

[b1] Michels B., Adamczyk F. & Koch J. Retrofit of a flue gas heat recovery system at the mehrum power plant. VGB PowerTech 84, 122–128 (2004).

[b2] Johnson I., Choate W. T. & Davidson A. *Waste heat recovery: technology and opportunities in U.S. industry*. *Technical report* (2008) Available at: http://www.osti.gov/scitech/biblio/1218716/ (Accessed: 9^th^ December 2015).

[b3] Osakabe M., Horiki S. & Hanaki Y. Prediction and performance of compact heat exchanger with small diameter tubes for latent heat recovery. J. Environ. Eng. 4, 36–46 (2009).

[b4] Jeong K., Kessen M. J., Bilirgen H. & Levy E. K. Analytical modeling of water condensation in condensing heat exchanger. Int. J. Heat Mass Transfer 53, 2361–2368 (2010).

[b5] Shi X. J., Che D. F., Agnew B. & Gao J. M. An investigation of the performance of compact heat exchanger for latent heat recovery from exhaust flue gases. Int. J. Heat Mass Transfer 54, 606–615 (2011).

[b6] Tang G. H., Hu H. W., Zhuang Z. N. & Tao W. Q. Film condensation heat transfer on a horizontal tube in presence of a noncondensable gas. Appl. Thermal Eng. 36, 414–425 (2012).

[b7] Ma X. H. . Convection condensation heat transfer of steam-air mixture with heat pipe heat exchanger. Heat Transfer Eng. 35, 600–609 (2014).

[b8] Folkedahl B. C., Weber G. F. & Collings M. E. W*ater extraction from coal-fired power plant flue gas. Technical report*. (2006) Available at: http://www.osti.gov/scitech/biblio/927112 (Accessed: 4^th^ July 2015).

[b9] Alkhudhiri A., Darwish N. & Hilal N. Membrane distillation: A comprehensive review. Desalination 287, 2–18 (2012).

[b10] Khraisheh M., Benyahia F. & Adham S. Industrial case studies in the petrochemical and gas industry in Qatar for the utilization of industrial waste heat for the production of fresh water by membrane desalination. Desalin. Water Treat. 51, 1769–1775 (2013).

[b11] Manawi Y. M., Khraisheh M., Fard A. K., Benyahia F. & Adham S. Effect of operational parameters on distillate flux in direct contact membrane distillation (DCMD): Comparison between experimental and model predicted performance. Desalination 336, 110–120 (2014).

[b12] Baker R. W. Future directions of membrane gas separation technology. Ind. Eng. Chem. Res. 41, 1393–1411(2002).

[b13] Sijbesma H. . Flue gas dehydration using polymer membranes. J. Membr. Sci. 313, 263–276 (2008).

[b14] Metz S. J., Van de Ven W. J. C., Potreck J., Mulder M. H. V. & Wessling M. Transport of water vapor and inert gas mixtures through highly selective and highly permeable polymer membranes. J. Membr. Sci. 251, 29–41 (2005).

[b15] Macedonio F., Brunetti A., Barbieri G. & Drioli E. Membrane condenser as a new technology for water recovery from humidified “waste” gaseous streams. Ind. Eng. Chem. Res. 52, 1160–1167 (2013).

[b16] Drioli E. . ECTFE membrane preparation for recovery of humidified gas streams using membrane condenser. React. Funct. Polym. 79, 1–7 (2014).

[b17] Brunetti A. . Waste gaseous streams: from environmental issue to source of water by using membrane condensers. CLEAN Soil Air Water 42, 1145–1153 (2014).

[b18] Zhang L. Z., Zhu D. S., Deng X. H. & Hua B. Thermodynamic modeling of a novel air dehumidification system. Energ. Buildings 37, 279–286 (2005). 10.1016/j.enbuild.2004.06.019 PMC7126610 32288121

[b19] Wang D. *Transport membrane condenser for water and energy recovery from power plant flue gas. Technical report*. (2012) Available at: http://www.osti.gov/scitech/biblio/1064416/ (Accessed: 23^th^ November 2013).

[b20] Wang D. X., Bao A. N., Kunc W. & Liss W. Coal power plant flue gas waste heat and water recovery. Appl. Energy 91, 341–348 (2012).

[b21] Bao A., Wang D. & Lin C. X. Nanoporous membrane tube condensing heat transfer enhancement study. Int. J. Heat Mass Transfer 84, 456–462 (2015).

[b22] Yan S., Zhao S., Wardhaugh L. & Feron P. H. M. Innovative use of membrane contactor as condenser for heat recovery in carbon capture. Environ. Sci. Technol. 49, 2532–2540 (2015). 25590169 10.1021/es504526s

[b23] Wang T. T., Yue M. W., Qi H., Feron P. H. M. & Zhao S. F. Transport membrane condenser for water and heat recovery from gaseous streams: Performance evaluation. J. Membr. Sci. 484, 10–17 (2015).

[b24] Macedonio F., Cersosimo M., Brunetti A., Barbieri G. & Drioli E. Water recovery from humidified waste gas streams: Quality control using membrane condenser technology. Chem. Eng. Process. Process Intensif. 86, 196–203 (2014).

[b25] Horikawa T., Do D. D. & Nicholson D. Capillary condensation of adsorbates in porous materials. Adv. Colloid Interface Sci. 169, 40–58 (2011). 21937014 10.1016/j.cis.2011.08.003

[b26] Uhlhorn R. J. R., Keizer K. & Burggraaf A. J. Gas transport and separation with ceramic membranes. Part I. Multilayer diffusion and capillary condensation. J. Membr. Sci. 66, 259–269 (1992).

[b27] Gregg S. J. & Sing K. S. W. In Adsorption Surface Area and Porosity 2^nd^ edn 121 (Academic Press, 1982).

[b28] Plimpton S. Fast parallel algorithms for short-range molecular dynamics. J. Comput. Phys. 117, 1–19 (1995).

[b29] Molinero V. & Moore E. B. J. Water modeled as an intermediate element between carbon and silicon. Phys. Chem. B 113, 4008–4016 (2009). 10.1021/jp805227c 18956896

[b30] De La Llave E., Molinero V. & Scherlis D. A. J. Water filling of hydrophilic nanopores. Chem. Phys. 133, 034513 (2010). 10.1063/1.3462964 20649343

[b31] Stillinger F. H. & Weber T. A. Computer simulation of local order in condensed phases of silicon. Phys. Rev. B 31, 5262 (1985). 10.1103/physrevb.31.5262 9936488

[b32] Krishnaswamy S., Wang H. S. & Rose J. W. Condensation from gas-vapour mixtures in small non-circular tubes. Int. J. Heat Mass Transfer 49, 1731–1737 (2006).

